# Knockdown of lactate dehydrogenase by adeno‐associated virus‐delivered CRISPR/Cas9 system alleviates primary hyperoxaluria type 1

**DOI:** 10.1002/ctm2.261

**Published:** 2020-12-21

**Authors:** Rui Zheng, Xiaoliang Fang, Xi Chen, Yunteng Huang, Guofeng Xu, Lei He, Yueyan Li, Xuran Niu, Lei Yang, Liren Wang, Dali Li, Hongquan Geng

**Affiliations:** ^1^ Department of Pediatric Urology Xinhua Hospital, Shanghai Jiao Tong University School of Medicine Shanghai China; ^2^ Children's Stone Treatment Center, National Health and Family Planning Commission of the People's Republic of China Shanghai China; ^3^ Shanghai Key Laboratory of Regulatory Biology Institute of Biomedical Sciences and School of Life Sciences East China Normal University Shanghai China; ^4^ Department of Urology Xinhua Hospital, Shanghai Jiao Tong University School of Medicine Shanghai China

**Keywords:** adeno‐associated virus, CRISPR/Cas9, gene therapy, lactate dehydrogenase, primary hyperoxaluria type 1

## Abstract

**Background:**

Primary hyperoxaluria type 1 (PH1) is a rare genetic disorder caused by endogenous overproduction of hepatic oxalate, leading to hyperoxaluria, recurrent calcium oxalate kidney stones, and end‐stage renal disease. Lactate dehydrogenase (LDH) is an ideal target for diminishing oxalate production as it is responsible for glyoxylate to oxalate conversion in the liver, the last step of oxalate metabolism. Here, we investigated the therapeutic efficacy and potential side effects of clustered regularly interspaced short palindromic repeats (CRISPR)/Cas9 technology to ameliorate PH1 via specifically disrupting the hepatic LDH.

**Methods:**

Pheochromocytoma (PC12) cells were used to assess the efficacy of cleavage of single‐guide RNAs in vitro. PH1 neonatal rats were injected with a single administration of adeno‐associated virus to deliver the CRISPR/Cas9 system that targeted LDH. Three weeks after injection, a liver biopsy was performed to detect LDH expression, liver injury, and liver metabolomics. Urinary oxalate was regularly monitored, and renal calcium oxalate deposition was evaluated after 4 weeks of 0.5% ethylene glycol challenge. After 6 months of treatment, animals were euthanized, and ex‐liver organs were harvested for toxicity analysis.

**Results:**

The *Ldha* gene was specifically knocked out in 20% of the liver cells of PH1 rats in the treatment group, leading to a 50% lower LDH expression than that in the control group. Compared to the control groups, urinary oxalate levels were significantly decreased, and renal calcium oxalate precipitation was largely mitigated in the treatment group throughout the entire 6‐month study period. While no CRISPR/Cas9‐associated off‐target edits or hepatotoxicity were detected, we observed mild metabolic changes in the liver tricarboxylic acid (TCA) and glycolysis pathways.

**Conclusions:**

CRISPR/Cas9‐mediated LDH disruption may represent an applicable new strategy for alleviating PH1 for its long‐lasting effect and low editorial efficiency requirements.

## Graphical abstract

CRISPR/Cas9 technology provides novel approaches for inherited monogenic diseases by targeting non‐essential enzymes. Using a rat model, we demonstrate that the AAV‐delivered CRISPR/Cas9 system is an effective and safe platform for knocking down lactate dehydrogenase and alleviating the life‐threatening primary hyperoxaluria type 1.

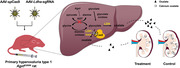

## 1 BACKGROUND

Primary hyperoxaluria type 1 (PH1; OMIM #259900), the most frequent and devastating subtype of PH,^1^ is characterized by a high level of urinary oxalate and recurrent urinary stones. PH1 is an autosomal recessive disease caused by the mutations in the gene‐encoding alanine‐glyoxylate aminotransferase (AGT) (EC 2.6.1.44), which is highly expressed in hepatocytes and primarily catalyzes glyoxylate into glycine.^2^, ^3^ Mutations in *AGXT* can result in AGT mistargeting, low catalytic activity, and reduced or abolished AGT expression. These, in turn, can lead to excessive accumulation of glyoxylate, which is converted into oxalate under the catalysis of lactate dehydrogenase (LDH) (EC 1.1.1.27) to cause recurrent urinary stones, loss of renal function, and ultimately renal failure.^1^, ^2^, ^4^ Patients with PH1 have a high risk of debilitation and end‐stage renal disease (ESRD) before adulthood, with 57% of the patients affected by 40 years of age.^3^ Moreover, as the condition progresses, a simultaneous kidney and liver transplantation becomes the only curative treatment option for most patients with PH1, which is expensive and challenging to implement.^5^ Hence, novel, efficient, and permanent treatment strategies are urgently needed to treat these patients.

Recent therapeutic advances involve targeting PH1 at the genetic and molecular level through substrate reduction therapy (SRT), which alleviates PH1 through inactivation of either glycolate oxidase (GO) (EC 1.1.3.15) that produces glyoxylate or LDH that catalyzes glyoxylate into oxalate.^6^, ^7^, ^8^, ^9^, ^10^ By comparing the effect of targeting LDH versus GO through RNA interference (RNAi), it has been demonstrated that compared with GO, less reduction of LDH protein was required to reduce oxalate production.^9^ Furthermore, a phase I clinical trial of LDH inhibition has shown encouraging preliminary results (NCT03392896). These findings indicate that LDH is an ideal therapeutic target for PH1. However, LDH‐inhibition therapies based on RNAi require repeated administrations, and the long‐term therapeutic effect remains unknown. Thus, it is important to find novel therapeutic approaches that are capable of curing PH1 permanently.

The clustered regularly interspaced short palindromic repeats (CRISPR)/Cas9 system is a mature and powerful genome‐editing tool,^11^, ^12^ which could generate insertions and deletions (indels) in the genome to disrupt LDH efficiently. Currently, adeno‐associated virus (AAV), the most promising in vivo delivery vector, is being combined with genome‐editing tools to treat various genetic disorders.^13^, ^14^, ^15^, ^16^, ^17^ Therefore, we hypothesized that disrupting LDH via the CRISPR/Cas9 system may present a better alternative treatment for patients with PH1 because of its permanent effect and the fact that a complete elimination of LDH is not required for the treatment of PH1.

In this study, we show that through the CRISPR/Cas9 system, a sustained reduction of LDH protein could be achieved in a PH1 rat model,^18^ leading to substantial reductions in oxalate production in PH1 rats. Additionally, we detected mild metabolic changes in the liver tricarboxylic acid (TCA) and glycolysis pathways in the treated rats at the end point of this study, which has not been carefully evaluated in previous reports.^19^, ^20^, ^21^, ^22^, ^23^ Though further research is required to validate the long‐term safety of the treatment, our results suggest that in vivo gene editing through CRISPR/Cas9‐mediated LDH disruption may be a promising therapeutic approach for patients with PH1.

## 2 MATERIALS AND METHODS

### 2.1 Vectors design

Two AAV vectors were constructed: Cas9 vector containing the liver‐specific promotor‐1 (LP1) promotor and *Streptococcus pyrogens* Cas9 (*sp*Cas9) sequence, and single‐guide RNA (sgRNA) vector‐containing U6 promotor, sgRNA scaffold, and green fluorescent protein (GFP) sequence. We used the Benchling software (www.benchling.com) to select sgRNAs targeting exonic regions of the *Ldha* gene. We cloned annealed oligonucleotides (Biosune, China) into the *Bbs*I site of the sgRNA vector.

### 2.2 Cell culture and sgRNA selection

The Pheochromocytoma (PC12) cells (American Type Culture Collection [ATCC], VA) were cultured in a commercial growth medium (RPMI 1640 supplemented with 10% fetal bovine serum, 1% penicillin, and 1% streptomycin). Approximately 2 × 10^5^ cells were cotransfected with 0.5 μg of Cas9 vectors and 0.5 μg of sgRNA vectors in each well of a 24‐well plate. The media were replaced every 24 hours, and the GFP cells were collected via flow cytometry (FACS Calibur, BD) 72 hours after transfection. Genomic DNA was isolated with an extraction kit (Tiangen, China). DNA fragments were amplified (Table S1) and sequenced at the company (Biosune, China).

The single‐strand annealing (SSA) luciferase assay was also performed to test the activity of sgRNAs.^24^, ^25^, ^26^ Briefly, we generated a luciferase reporter plasmid that contained a luciferase gene, which was interrupted by stop codons, a DNA fragment of the rat *Ldha* gene, and flanking complementary repeat sequences. Upon cleavage of the target site by Cas9 nucleases, the complementary repeats restored the expression of the luciferase gene. The reporter plasmid, the Cas9, and sgRNA vector were cotransfected into HEK293T cells (ATCC, VA) and cultured for 48 hours. We used a luciferase detection system (Promega, CA) to determine the luciferase activity.

### 2.3 AAV8 production and administration

The procedure of AAV production was described in our previous study.^18^ Briefly, AAV Rep2‐Cap8 and an adeno helper plasmid were cotransfected with the Cas9 vector or sgRNA vector in HEK293T cells using polyethylenimine (No. 24765‐2, Polysciences, PA). Sixty hours later, the cells were lysed and digested with Benzonase (No. 70746‐3, Merck‐Novagen, Germany), followed by freeze‐thaw cycles. Then, an iodixanol gradient ultracentrifugation was carried out to purify the viral vectors. The viral vectors were then concentrated 10‐fold to ∼1 mL using 15 mL, 100 kDa molecular weight cut‐off filters (Merck Millipore, Germany). AAV titration was determined by quantitative PCR (qPCR) method using SYBR Premix Ex Taq (Yeason, China). Newborn (postnatal day 7) male PH1 pups were injected with both 1 × 10^12^ vector genome (vg) of AAV8‐*sp*Cas9 and 1 × 10^12^ vg of AAV8‐sgRNA‐EGFP via the tail at a final volume of 200 μL. Each control newborn rat was injected with 1 × 10^12^ vg of AAV8‐sgRNA‐EGFP or with PBS only.

### 2.4 Animal experiments

All animal experiments conformed to the regulations drafted by the Institutional Animal Care and Use Committee of the Ethics Committee of Xinhua Hospital Affiliated to Shanghai Jiao Tong University School of Medicine (XHEC‐F‐2017‐NSFC‐001) and the Shanghai Laboratory Animal Commission, and was permitted by the East China Normal University Public Platform for innovation (011). All *Agxt^D205N^* rats were genotyped as described,^18^ with age‐matched Sprague Dawley (SD) rats used as controls. Animals were kept in a pathogen‐free facility on a 12 hours/12 hours light/dark cycle with ad libitum access to standard chow and water.

Partial hepatectomy was performed 3 weeks after treatment to detect LDH expression, liver injury, and deep sequencing; the surgical procedure was carried out as previously reported.^18^ Rats were sacrificed 6 months after vector treatment, and liver, kidney, skeletal muscle, heart, spleen, brain, and lung tissues were harvested for analysis.

To test whether nephrocalcinosis was prevented, water with a 0.5% ethylene glycol (EG) (v/v) was given to four 6‐month‐old rats in each group for 4 weeks, during which time the rats were weighed weekly. Urine was collected 6 days after the EG challenge, and the rats were euthanized at the end of the fourth week for tissue harvest.

### 2.5 On‐target and off‐target mutagenesis analyses

The genomic DNA of rat tissues was extracted using the phenol‐chloroform method. As shown in Table S2, candidate off‐target sites were identified using the Benchling software (www.benchling.com). The genomic region was amplified and the amplicons were sent for deep sequencing analysis (Novogene, Beijing). To detect indels in *Ldha*, we also designed a pair of primers to amplify the relevant sequence and sent it for deep sequencing. All primers are listed in Table S1. Finally, the high‐throughput sequencing data were submitted to CRISPR software (http://www.rgenome.net/cas-analyzer/) to assess and characterize indels. The data were uploaded to the NCBI Sequence Read Archive (SRA) database with the accession number PRJNA646016.

### 2.6 Real‐time (RT)‐qPCR

RNA was isolated using RNAiso Plus (TaKaRa, Japan). Reverse transcription was carried out using PrimeScript RT Master Mix (RR036A, TaKaRa, Japan). RT‐qPCR was performed on a QuantStudio3 real‐time PCR system (Applied Biosystems, MA) to measure rat *Ldha*, *sp*Cas9, EGFP, and β‐actin levels. Primers are listed in Table S1.

### 2.7 Western blot analysis

Western blot analyses were performed on rat liver lysates, as described previously.^18^ LDH protein was probed with an anti‐LDH antibody (ab52488, Abcam, UK) diluted at 1:2000. Mouse anti‐β‐actin antibody diluted at 1:2000 (A5441, Sigma, Germany) was used to detect β‐actin. An Odyssey Infrared Imaging System (LI‐COR, NE) was used for imaging of the blots.

### 2.8 Sample collections and measurements

Blood was collected using retro‐orbital puncture of four rats from each group and sent for aspartate aminotransferase (AST), alanine aminotransferase (ALT), and total bilirubin (TBIL) detection in serum (Servicebio, China). Urine was collected over 24 hours using metabolic cages and acidified with hydrochloric acid before measurement. The oxalate concentrations were determined by ion‐exchange chromatography using a Dionex ICS‐5000 (Thermo Scientific, MA).

### 2.9 Histopathology

For frozen sections, rat liver tissues were embedded in OCT compound, and imaged using an inverted laser scanning microscope (TCS SP8, Leica, Germany). Liver and kidney samples were fixed with 4% paraformaldehyde overnight, embedded in paraffin, and sectioned at 4 μm thick. Hematoxylin and eosin (H&E) stainings were performed according to standard protocols. For IHC, 5% hydrogen peroxide was used to block endogenous peroxidase, followed by overnight incubation with anti‐LDH antibody (ab52488, Abcam) to analyze LDH expression, anti‐CD68 (Bio‐Rad, CA) to trace inflammation, or anti‐α‐SMA (Invitrogen, CA) to detect liver fibrosis at 1:500, 1:200, and 1:200 dilutions, respectively. The slides were then incubated with horseradish peroxidase (HRP)‐conjugated secondary antibodies at 1:5000 dilution for 1 hour. Detection of HRP was performed as described in the DAB detection kit (SK‐4100, Vector Labs, CA). To visualize CaOx deposition in sections of kidney samples, Pizzolato staining was carried out as described previously.^27^

### 2.10 Metabolite quantification and metabolomics

A total of 40 mg of liver sample was harvested from each rat; 50 μL ultra‐pure water and 10 μL 20 μM internal standards were added to the sample for homogenization. Then 390 μL precooled methanol/acetonitrile (1:1, v/v) was added to liver homogenate samples or to 40 μL plasma samples extracted from rats, followed by vortex mixing. The supernatant was harvested after precipitating the protein by centrifugation at 14 000 RCF at 4°C for 20 minutes. Quintuplicate samples were collected and sent for analysis by metabolon‐associated energy metabolism (Applied Protein Technology, China).

### 2.11 Statistics

Data are presented as means ± SD. Unpaired *t*‐tests were used to compare two groups (two‐tailed). The significant *P*‐values were denoted on the graphs, and a *P* < .05 was considered to be statistically significant.

## 3 RESULTS

### 3.1 Selection of sgRNA target sites

**FIGURE 1:**
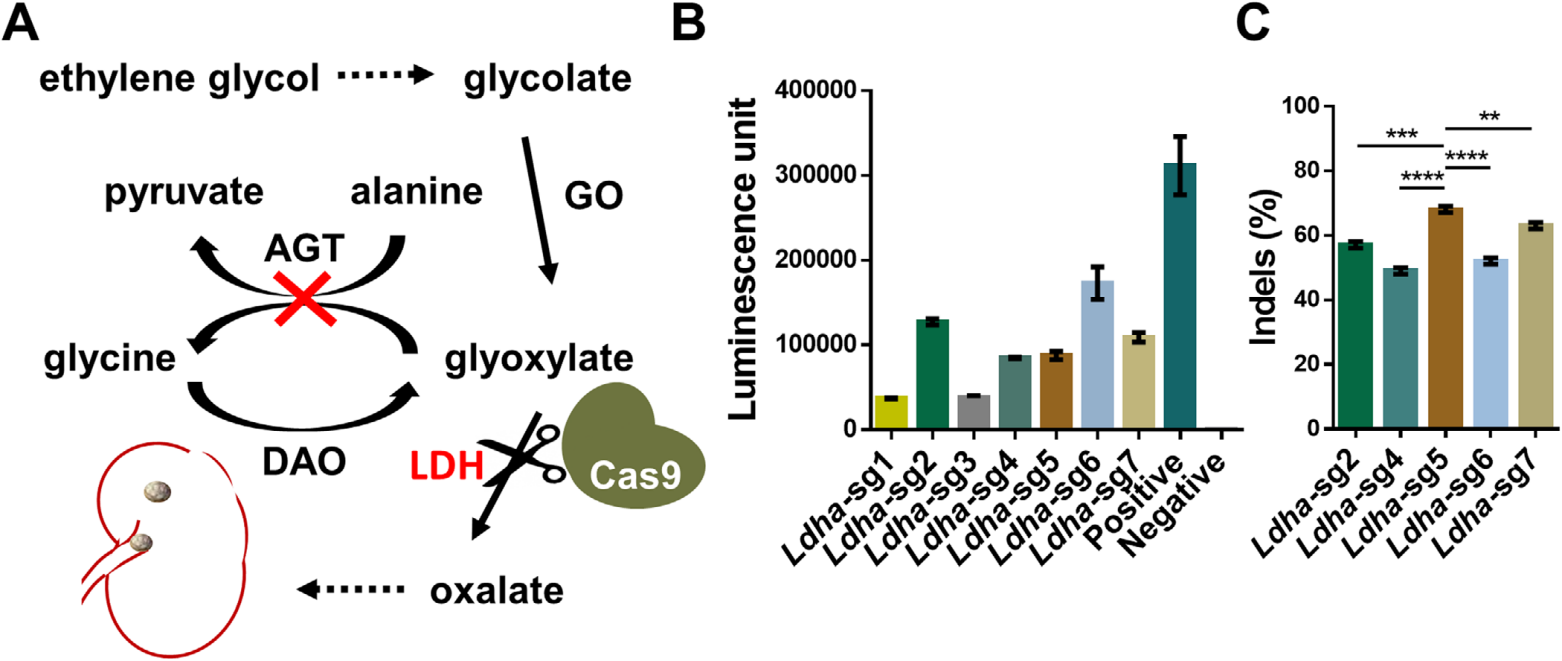
The mechanism for CRISPR/Cas9‐mediated PH1 treatment and sgRNA screening. A, Schematic of PH1 treatment through LDH disruption. B, In vitro single‐guide (sg) RNA screening using single‐strand annealing (SSA) luciferase assay. C, In vitro sgRNA screening using Sanger sequencing analysis. Results: mean ± SD. **P < .01, ***P < .001, ****P < .0001. AGT, alanine glyoxylate aminotransferase; DAO, diamine oxidase; GO, glycolate oxidase; LDH, lactic dehydrogenase

To select a highly active sgRNA targeting the *Ldha* that could reduce oxalate production (Figure 1A), a vector containing *sp*Cas9, sgRNA, and GFP sequences was generated. According to the predicted high on‐target and low off‐target probabilities, seven sgRNAs targeting the coding region of *Ldha* were selected and cloned into the above vector. In the preliminary screening, the SSA luciferase reporter assay pointed to five alternative sgRNAs with considerable activity (Figure 1B). These five vectors were selected and transfected into a PC12 cell line, and the vectors’ targeting efficiency was tested by Sanger sequencing. Among all designed sgRNAs, we found that sgRNA5 had the highest targeting efficiency and therefore was used for in vivo experiments (Figure 1C).

### 3.2 AAV‐CRISPR/Cas9 treatment partially disrupts hepatic LDH expression in *Agxt^D205N^* rats

Due to the limited size capacity (< 4.7 kb) of the AAV vector for gene delivery, a dual AAV system was used: one carrying the Cas9 with a LP1 promoter (named AAV‐Cas9) and the other carrying the sgRNA and GFP, driven by a U6 and a CMV promoter, respectively (named AAV‐sgRNA). AAV serotype 8 was used as it could precisely and efficiently transduce neonatal hepatocytes.

**FIGURE 2:**
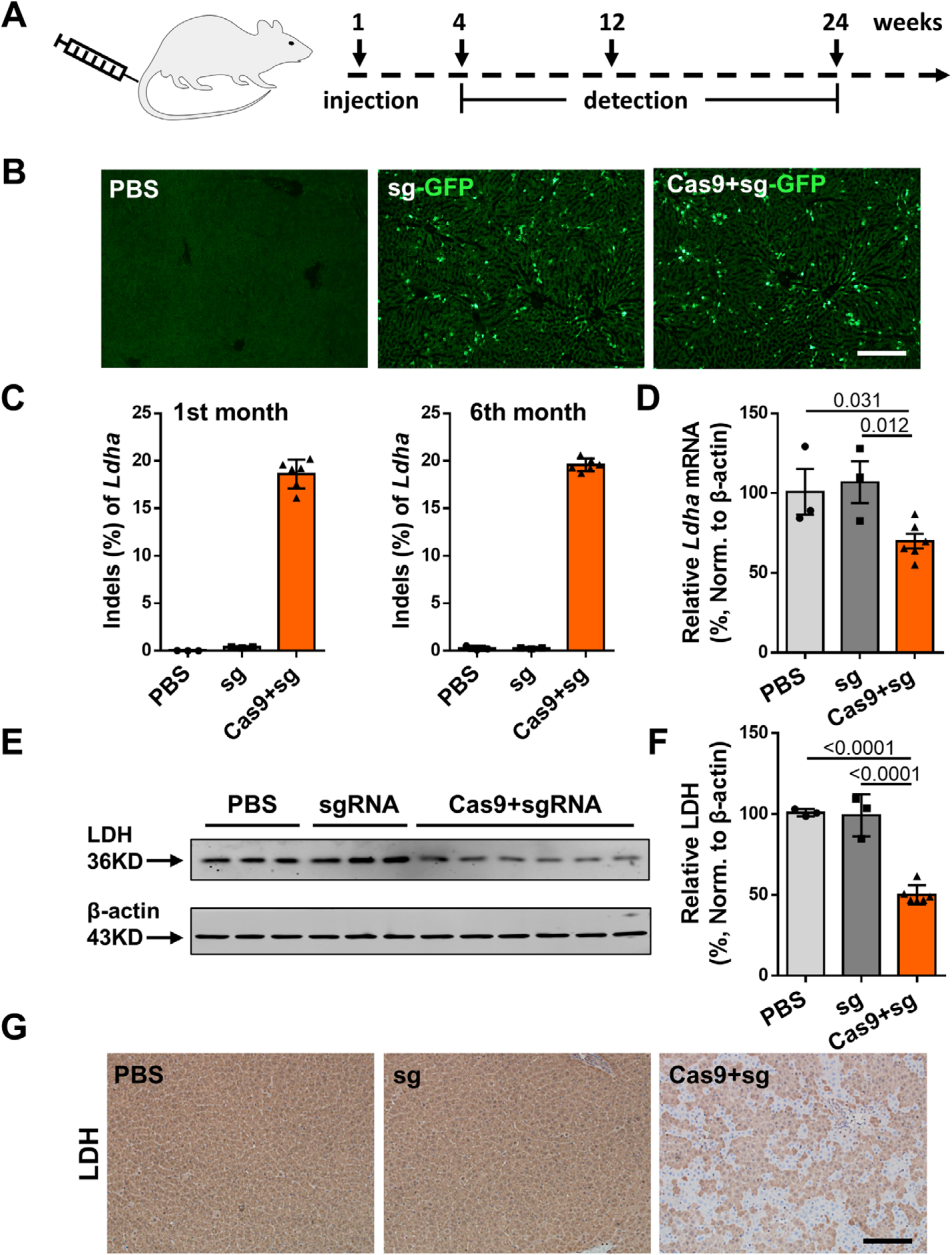
CRISPR/Cas9‐mediated in vivo knockdown of *Ldha* expression. A, Schematic figure showing experimental time points for treatment and analysis. B, Frozen sections of livers from 4‐week‐old *Agxt^D205N^* rats injected with PBS, AAV‐sgRNA, or dual AAV‐Cas9 and AAV‐sgRNA under excitation wavelength (465 nm). Bar = 200 μm. C, Editing efficiencies of *Ldha* in *Agxt^D205N^* rats 1 month (left) and 6 months (right) after injection with PBS (n = 3), AAV‐sgRNA (n = 3), or dual AAV‐Cas9 and AAV‐sgRNA (n = 6). D, Relative mRNA expression of *Ldha* gene in liver tissue from 4‐week‐old *Agxt^D205N^* rats injected with PBS (n = 3), AAV‐sgRNA (n = 3), or dual AAV‐Cas9 and AAV‐sgRNA (n = 6). E, Western blot analysis and quantification (F) of LDH expression in liver tissue from 4‐week‐old *Agxt^D205N^* rats injected with PBS (n = 3), AAV‐sgRNA (n = 3), or dual AAV‐Cas9 and AAV‐sgRNA (n = 6). G, LDH IHC staining of liver tissue sections from *Agxt^D205N^* rats after 4 weeks of treatment. Bar = 100 μm. Results: mean ± SD. Significant *P‐*values are denoted above the bars. Norm., normalized

To provide early intervention, in the treatment group, we delivered 5 × 10^11^ vg of AAV‐Cas9 and 1 × 10^12^ vg of AAV‐sg‐GFP per male rat via tail vein injection on the 7th day after birth (Figure 2A). As controls, we set up a second group injected with 1 × 10^12^ vg of AAV‐sgRNA without Cas9 (called the sgRNA group) and a third group injected with PBS (called the mock group) at the same time.

To visualize the viral transduction, we analyzed the livers of 4‐week‐old rats for GFP fluorescence and found that hepatocytes in AAV‐injected animals (the treatment and the sgRNA groups) were partially GFP‐positive (Figure 2B). To assess CRISPR/Cas9‐mediated gene‐editing precision, we analyzed on‐target and off‐target sites of *Ldha*‐targeting sgRNA. Indels were detected in 18.6 ± 0.6% of the *Ldha* alleles from the treatment group, and this editing efficiency was steady during the 6‐month follow‐up period (Figure 2C). Characterization of the indel patterns generated by CRISPR/Cas9 is shown in Figures S1 and S2. Then we amplified the potential off‐target sites of sgRNA, and found the indel frequencies were at the background levels (Table S2). We also analyzed the on‐target sites of rats from the sgRNA group and the mock group and found no apparent indels in either of the control groups.

Next, we examined the expression of *Ldha* at the transcription and translation levels. RT‐qPCR results showed that *Ldha* mRNA expression in the treatment group was 70.0 ± 0.1% of that in the mock group (Figure 2D). Consistently, the LDH protein level in the treatment group was reduced to 50.1 ± 2.4% of the control levels in the mock group, and there was no significant decrease in the LDH protein level in the sgRNA group (Figure 2E,F). Additional evidence of LDH expression knockdown was supported by immunostaining data of liver sections. These data reveal that LDH expression was absent in the partial hepatocytes of the treatment group, whereas the LDH protein was present in all liver cells from both of the control groups (Figure 2G).

Together, these results indicate that AAV‐CRISPR/Cas9‐mediated in vivo gene editing could knockdown the expression of LDH protein in *Agxt^D205N^* rats.

### 3.3 Partial LDH disruption ameliorates PH1 phenotypes in *Agxt^D205N^* rats

**FIGURE 3:**
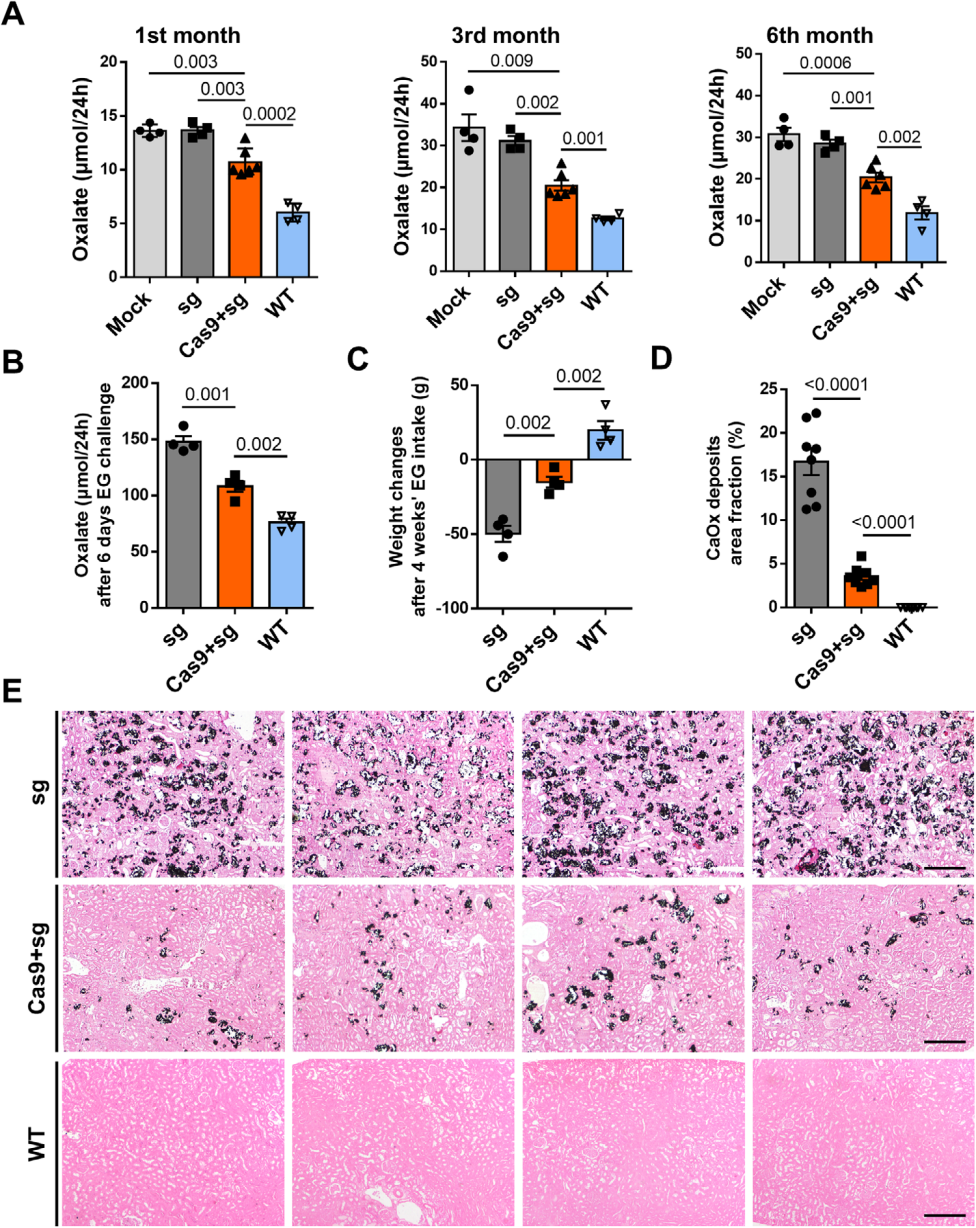
*Ldha* disruption alleviated the PH1 phenotypes in *Agxt^D205N^* rats. A, Twenty‐four‐hour urinary oxalate levels were measured in the 1st, 3rd, and 6th months of the experiment after a single dose of PBS (n = 4), AAV‐sgRNA (n = 4), or dual AAV‐Cas9 and AAV‐sgRNA (n = 6) was injected to neonatal *Agxt^D205N^* rats. Wild‐type (WT) rats (n = 4) of the same age were used as controls. B, Quantification of 24‐hour urinary oxalate levels after 6 days of 0.5% EG challenge (n = 4 in each group). C, Weight changes in rats after 4 weeks of 0.5% EG challenge (n = 4 in each group). D, Quantification of CaOx areas (%) in renal sections (n = 8 in each group). E, Representative Pizzolato staining of renal sections from *Agxt^D205N^* rats sacrificed 5 months after treatment with PBS (n = 4), AAV‐sgRNA (n = 4), or dual AAV‐Cas9 and AAV‐sgRNA (n = 4), and 1 month after 0.5% EG challenge. Bar = 200 μm. Results: mean ± SD. Significant *P‐*values are denoted above the bars. EG, ethylene glycol

To assess the efficacy of AAV‐CRISPR/Cas9‐mediated LDH disruption for the treatment of PH1, we analyzed the hyperoxaluria phenotype and kidney CaOx deposition in four groups of rats. Since PH1 rats become hyperoxaluric within 1 month, we analyzed oxaluria levels from 1 month of age onwards. Rats injected with dual AAV vectors had 21.7%, 40.3%, and 33.6% lower levels of urinary oxalate than the PH1 control groups in the 1st, 3rd, and 6th months of the experiment, respectively, though the levels were slightly higher than those of wild‐type (WT) animals (Figure 3A). We then added EG, a precursor of glyoxylate, to the drinking water to increase oxaluria. Compared to the WT groups, the urinary oxalate levels on day 6 of EG challenge were significantly higher in the treatment group: 147.9 ± 4.9 μmol/24 hours in the sgRNA group and 108.2 ± 4.9 μmol/24 hours in the treatment group compared to 76.1 ± 3.4 μmol/24 hours in the WT group (Figure 3B). On average, the bodyweight of the treatment group increased progressively over 4 weeks of EG challenge, which was second to that of WT rats; the mock group on the other hand underwent severe weight loss (Figure 3C). Pizzalato's staining and quantification of the stained CaOx in the renal cortex showed that the renal deposition of CaOx was significantly reduced in the treatment group (3.6 ± 0.4% area fraction) compared with the sgRNA group (16.7 ± 1.5% area fraction) (Figure 3D). At high magnification, in the sgRNA group, we observed the glomerular deformation and a wide range of dilatation of renal tubules, with a small amount of secretion in the tubular lumen. A representative image of the renal cortex from each animal is shown in Figure 3E. Similarly, visualization of the stained CaOx in renal medulla tissue showed significantly lower staining compared to that in the control groups (Figure S3). The staining also indicates that the CaOx deposition in the medullary area was much milder than that in cortical regions. Therefore, we conclude that our gene‐editing method was efficacious in treating hyperoxaluria phenotypes in the PH1 rat model.

### 3.4 AAV‐CRISPR/Cas9 system does not induce liver injury

**FIGURE 4:**
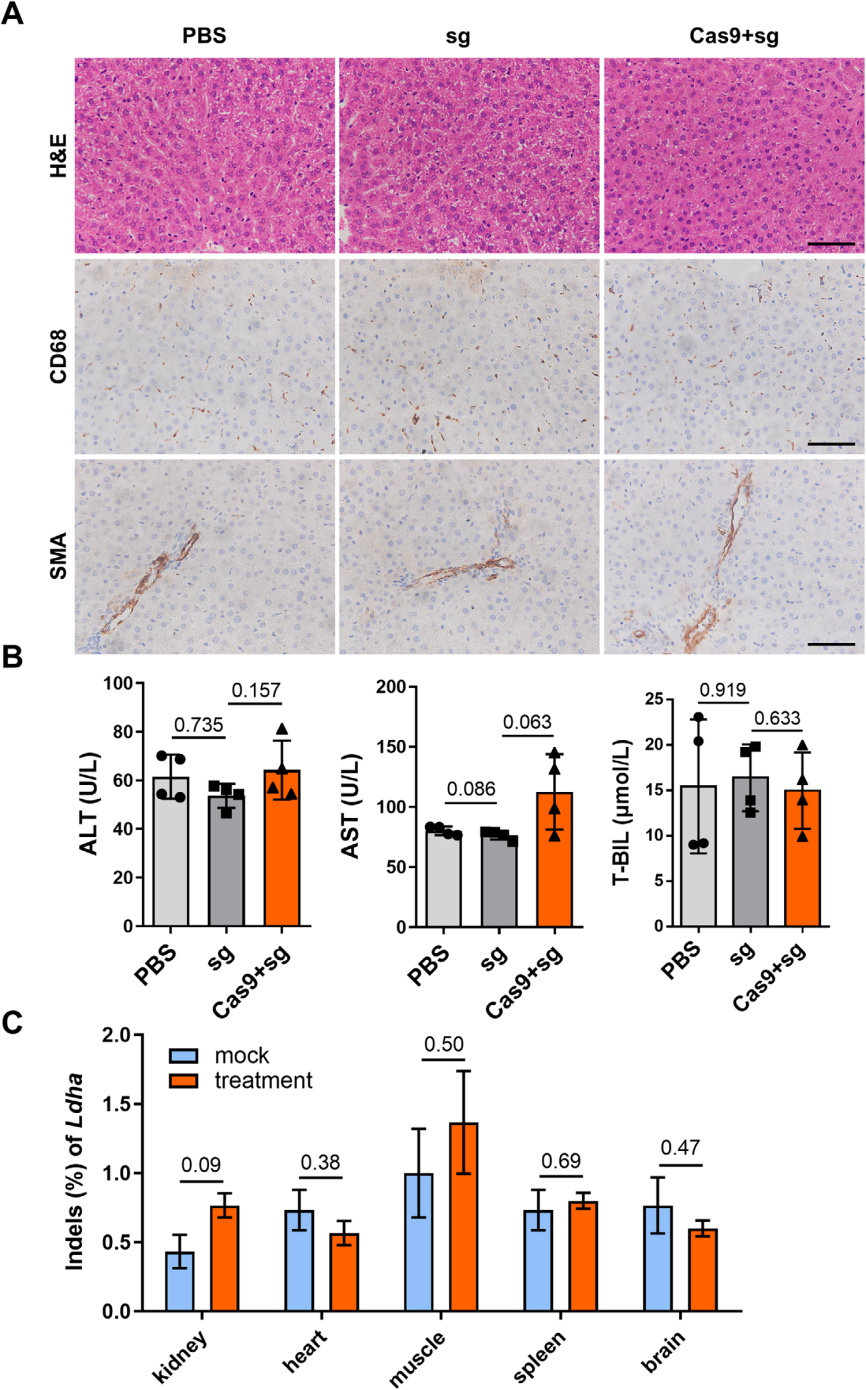
No liver or ex‐liver toxicity was detected in the AAV‐delivered CRISPR/Cas9 system. A, Representative hematoxylin and eosin (H&E) staining, cluster of differentiation (CD)68 and smooth muscle actin (SMA) immunohistochemical staining of liver sections from *Agxt^D205N^* rats 3 weeks after treatment with PBS (n = 4), AAV‐sgRNA (n = 4), or dual AAV‐Cas9 and AAV‐sgRNA (n = 6). Bar = 50 μm. B, Serum aspartate aminotransferase (AST), alanine aminotransferase (ALT), and total bilirubin (TBIL) levels in *Agxt^D205N^* rats 3 weeks after treatment (n = 4 in each group). C, The average percentage of variants in the *Ldha* on‐target region in different tissues. Results: mean ± SD. Significant *P‐*values are denoted above the bars

Apart from the CRISPR/Cas9‐associated off‐target analysis mentioned previously to rule out the genotoxic effect, we detected no signs of potential hepatotoxicity when we examined liver inflammation and serum biochemistry 3 weeks after injection. Histological analysis of liver sections by H&E staining showed unchanged histology and normal inflammatory cell infiltration (Figure 4A). Immunostaining on liver tissue samples for macrophages marker CD68 and smooth muscle cells marker SMA showed a normal degree of macrophage distribution and collagen deposition in all groups (Figure 4A). Consistently, we did not detect significant elevation in AST, ALT, and TBIL levels 3 weeks after injection (Figure 4B). Moreover, we did not find a significantly higher indel rate in the treatment groups compared to controls in any of the tested ex‐liver tissues (Figure 4C). These data corroborate the liver‐specificity of our therapeutic approach, and support the notion that LDH disruption mediated by AAV‐delivered CRISPR/Cas9 yielded no liver injury.

### 3.5 LDH knockdown leads to slight changes in liver metabolites

**FIGURE 5:**
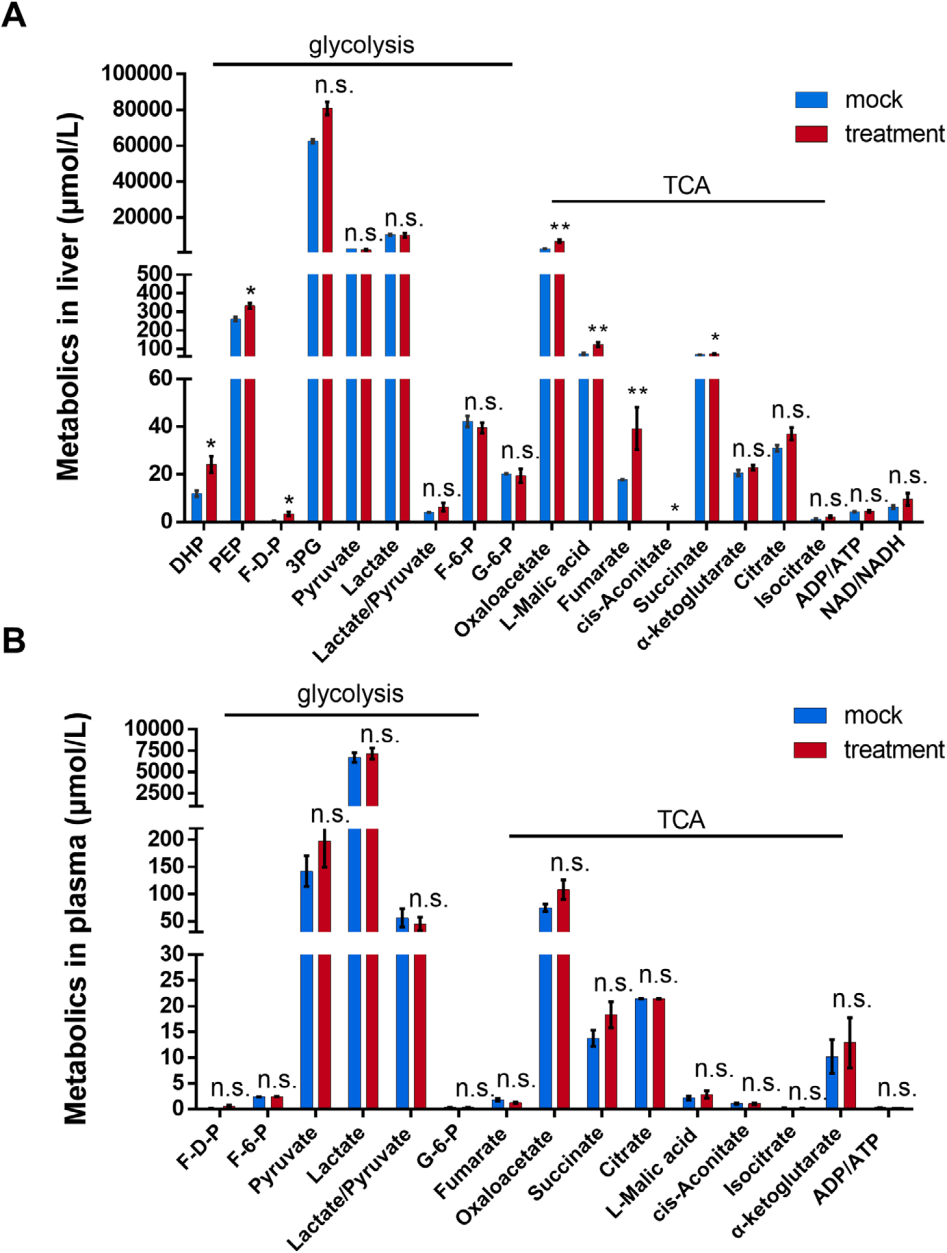
Change in metabolite levels after *Ldha* knockdown. Intermediate metabolites analysis of glycolysis, tricarboxylic acid cycle (TCA), and oxidative phosphorylation in the liver (A) and plasma (B) extracts from PH1 rats (n = 4 in each group) 3 weeks after AAV‐sgRNA or dual AAV‐Cas9 and AAV‐sgRNA injection. Results: mean ± SD; n.s., not significant, *P < .05, **P < .01. 3PG, 3‐phospho‐d‐glycerate; ADP, adenosine diphosphate; ATP, adenosine triphosphate; DHP, dihydroxyacetone phosphate; F‐6‐P, beta‐d‐fructose 6‐phosphate; F‐D‐P, d‐fructose 1,6‐bisphosphate; G‐6‐P, d‐glucose 6‐phosphate; NAD, nicotinamide adenine dinucleotide; NADH, nicotinamide adenine dinucleotide reduced; PEP, phosphoenolpyruvate

LDH is a tetramer that consists of LDH‐M and LDH‐H forms, encoded by *LDHA* and *LDHB*, respectively. In hepatocytes, LDH‐M is the only isomer form and the LDH isozyme of the liver consists of four LDH‐M subunits.^28^ In this study, we confirmed that *Ldha* but not *Ldhb* is expressed in the rat liver (Figure S4A), and that *Ldhb* expression was not affected by *Ldha* knockdown (Figure S4B). Other than participating in oxalate metabolism, in the liver, LDH also plays a vital role in the interconversion between lactate and pyruvate, which primarily acts in carbohydrate metabolism. Our results show that rats with partial disruption of LDH had similar liver pyruvate and lactate levels to the control rats, and we obtained similar results from plasma samples (Figure 5A,B). However, slight increases in most TCA‐cycle intermediates, such as oxaloacetate, l‐malic acid, fumarate, *cis*‐aconitate, and succinate, were observed in the liver extracts but not in the plasma of the treatment group. These data suggest that liver TCA intermediates may be more sensitive than pyruvate and lactate for monitoring the consequences of LDH disruption, especially under aerobic conditions. Similar to the changes in TCA intermediates, we also noticed that decreased LDH levels led to a slight but significant increase of liver phosphoenolpyruvate (1.2‐fold); this caused a more drastic elevation of liver d‐fructose‐1,6‐bisphosphate (4.7‐fold) and dihydroxyacetone phosphate (2.0‐fold), suggesting an increased gluconeogenesis flux (Figure 5A). None of the changes described above were observed in plasma (Figure 5B). Nevertheless, the procedures of oxidative phosphorylation were dynamically stable because the ratios of NAD:NADH and ADP:ATP were not significantly altered in the liver.

## 4 DISCUSSION

We have recently reported a PH1 rat model that exhibited high urinary oxalate levels at an early age, highly mimicking PH1 in humans.^18^ In the present study, using the same rat model, we observed a sustained reduction of LDH after a single administration of AAV‐delivered CRISPR/Cas9 system that targets rat *Ldha*. Moreover, we achieved a long‐term reduction in endogenous oxalate synthesis and a decrease in kidney CaOx precipitation in the treated PH1 rats, demonstrating the therapeutic potentials of this approach.

We and others have previously demonstrated in both mouse and rat PH1 models that CRISPR/Cas9‐targeting GO is a feasible strategy for treating PH1 effectively.^18^, ^29^ However, LDH is also an ideal target as it plays an essential role in glyoxylate to oxalate conversion, which is the last step in oxalate production. It has been shown that a significant reduction of urinary oxalate could be achieved when siRNA dose levels caused only a 24% reduction in LDH protein expression.^9^, ^10^ By contrast, a more substantial decrease in GO protein (at least 50%) is required to elicit a significant reduction in urinary oxalate levels. In line with the above findings, our data show that a 20% indel rate in the *Ldha* gene led to a significant decrease (nearly 40%) in urinary oxalate level, though at a 1.5‐fold higher value than that in WT rats. In our PH1 rat model, at least a 30‐45% indel rate of *Hao1* was required to achieve similar efficacy.^18^ The efficiency of genome editing in liver might be underestimated, as studies have shown that the AAV cannot transfect nonparenchymal cells, which accounts for nearly half of the liver cells (we used total liver tissue for indel detection).^30^, ^31^ Accordingly, our data showed a more considerable reduction in the LDH protein level compared to the indel rate. More importantly, the editing efficiency was consistent during the entire duration of our experiment (6 months), and this suggests that the edited hepatocytes can self‐renew even when rats are treated at the neonatal stage that would undergo a tremendous organ growth period.

As the AAV‐CRISPR/Cas9‐mediated gene editing modifies genes permanently, the safety of *Ldha* knockout in hepatocytes is worth considering. On the one hand, the accuracy of genome editing requires careful testing. A comprehensive analysis of off‐target effects has demonstrated rare single‐nucleotide variants induced by an elaborately designed CRISPR‐Cas9 system,^32^ which is consistent with our results. However, large deletions, complex genomic rearrangements, and cancer‐related inactivation of the p53 pathway have been observed after genome editing.^33^, ^34^, ^35^ Other risks of genome editing include immunogenicity of bacterially derived proteins and pre‐existing antibodies against CRISPR components that may cause inflammation.^36^, ^37^ These issues can be controlled or avoided through various strategies but still need to be thoroughly addressed before clinical application.^38^, ^39^ On the other hand, the consequence of *Ldha* deletion should be evaluated in detail. Although hereditary LDHA‐deficient patients exhibit abnormal extrahepatic conditions, such as exertional myopathy and skin lesions,^19^, ^20^, ^23^ further research on hepatic carbohydrate metabolism is needed. Lai et al conducted a well‐designed study to demonstrate the efficacy of LDH inhibition and found no abnormalities in the extrahepatic tissues.^9^ However, circulating liver enzymes rather than metabolites in liver tissue were monitored in that study. In our AAV‐delivered CRISPR/Cas9 system, we did not observe any drastic changes in circulating carbohydrate intermediates or liver injury. We did, however, observe a slight but steady increase of some intermediates in TCA and gluconeogenesis pathways in liver tissues of the edited rats but no alteration in lactate and pyruvate levels, which probably contributed to the sufficient function of residual hepatic LDH completing the Cori cycle. Similar to our findings, Wood et al^10^ also reported significant metabolic changes in the liver TCA cycle and increased pyruvate levels and impacted ATP synthesis/consumption following high‐dose siRNA treatment. However, the researchers did not investigate the changes in the gluconeogenesis pathway. These results suggest that intermediates in TCA and gluconeogenesis pathways are sensitive markers, which should be combined with liver lactate and pyruvate levels to monitor the consequences of LDH disruption.

In conclusion, our data suggest that a modest knockdown of LDH may be suitable for simultaneously restricting oxalate synthesis and limiting metabolic changes in the liver. In this regard, future studies would give us a better understanding of the consequences of LDH knockdown. One exciting idea is to combine LDH disruption with GO disruption to further decrease oxalate excretion, to a near‐normal level, to avoid a progressive and unpredictable decline in kidney function. With the gene sequencing technology becoming more available at a lower cost, PH1 could be diagnosed earlier, which would allow earlier treatment via novel technologies such as genome editing.

## AUTHOR CONTRIBUTIONS

Hongquan Geng and Dali Li formulated and designed the project. Rui Zheng, Xiaoliang Fang, and Xi Chen devised and performed all experiments. Lei Yang, Yueyan Li, and Liren Wang assisted in SSA assay design and experiments. Liren Wang, Hongquan Geng, and Dali Li aided figure generation and manuscript preparation. Xuran Niu and Lei He contributed to animal experiments. Guofeng Xu and Yunteng Huang assisted in data interpretation. Rui Zheng and Lei Yang performed the statistical analysis. Rui Zheng, Xiaoliang Fang, and Xi Chen wrote the manuscript with input from all authors. Science and Technology Commission of Shanghai Municipality, Grant Numbers: 18XD1403100, 20140900201; National Natural Science Foundation of China, Grant Numbers: 81770702, 81670470; Shanghai Municipal Health and Family Planning Commission, Grant Number: 2017BR061

## CONFLICT OF INTEREST

The authors declare that there is no conflict of interest.

## Supporting information

Supporting Information

## Data Availability

The data that support the findings of this study are available from the corresponding author upon reasonable request.
