# Supplementary material for: Knockdown of lactate dehydrogenase by adeno‐associated virus‐delivered CRISPR/Cas9 system alleviates primary hyperoxaluria type 1
Source: Clin Transl Med. 2020 Dec 21;10(8):e261. doi: 10.1002/ctm2.261 (PMC7752156; doi:10.1002/ctm2.261)
Supplement: Supplementary file 1 — Supporting Information [file CTM2-10-e261-s001.pdf]

Supplemental Table 1. Primers and sgRNAs used in this study.

Primers for PCR amplification

**Primer ID**	**Primer sequence**	**Primer ID**	**Primer sequence**
Hitom-*Ldha*-on-F	GGAGTGAGTACGGTGTGCGAAGCTTTGCTGGGTAGGACAC	Hitom-*Ldha*-on-R	GAGTTGGATGCTGGATGGGATGTTCACGTTTCGCTGGACC
Hitom-*Ldha*-OT1-F	GGAGTGAGTACGGTGTGCTGGACTGTATTTCACAAAGTTCGG	Hitom-*Ldha*-OT1-R	GAGTTGGATGCTGGATGGTCTTCAGCATGGCAGCCTTT
Hitom-*Ldha*-OT2-F	GGAGTGAGTACGGTGTGCTCACGTTTCGCTGGACCAAA	Hitom-*Ldha*-OT2-R	GAGTTGGATGCTGGATGGATGATGGATCTCCAGCACGG
Hitom-*Ldha*-OT3-F	GGAGTGAGTACGGTGTGCTCACGTTTCGCTGGACCAAA	Hitom-*Ldha*-OT3-R	GAGTTGGATGCTGGATGGATGATGGATCTCCAGCACGG
Hitom-*Ldha*-OT4-F	GGAGTGAGTACGGTGTGCACATTTCCCTGGACCAAATTAAGC	Hitom-*Ldha*-OT4-R	GAGTTGGATGCTGGATGGGATGGATCTCCAGCATGGCA
Hitom-*Ldha*-OT5-F	GGAGTGAGTACGGTGTGCGCACTGTGGACTCTACTTCCC	Hitom-*Ldha*-OT5-R	GAGTTGGATGCTGGATGGAGCATGGCAGCCTTTTCCTT
Hitom-*Ldha*-OT6-F	GGAGTGAGTACGGTGTGCCACTGGACCAAATTGAGCCG	Hitom-*Ldha*-OT6-R	GAGTTGGATGCTGGATGGGATGGATCTCCAGCATGGCA
Hitom-*Ldha*-OT7-F	GGAGTGAGTACGGTGTGCAGCATGGCAGCCTTTTCCT	Hitom-*Ldha*-OT7-R	GAGTTGGATGCTGGATGGTGCACTGTGGACTGTATTTCA
Hitom-*Ldha*-OT8-F	GGAGTGAGTACGGTGTGCATTGAGTCGGCTCTCTCCCT	Hitom-*Ldha*-OT8-R	GAGTTGGATGCTGGATGGCTTGGCGATGAGCTTGCTTG
Ldha-qPCR-F	GGCATGGCTTGTGCCATCA	*Ldha*-qPCR-R	AGGCTGCCATGCTGAAGATC
Ldhb-qPCR-F	GTGTGGCTGACCTCATCGAA	*Ldhb*-qPCR-R	TGCCGTACATTCCCTTCACC
Rat-actin-F	GGGAAATCGTGCGTGACATT	Rat-actin-R	GCGGCAGTGGCCATCTC
Ldha-Exon3-F	CCATGGCAAGTCCTGAGAAGA	*Ldha*-Exon3-R	GGAACTTAAGCCTGACAGCAG

Ldha-Exon4-F	GAAGCTTTGCTGGGTAGGACAC	*Ldha*-Exon4-R	GATGTTCACGTTTCGCTGGACC
Ldha-Exon6-F	ACACCGTTAGGGAGCAGTTG	*Ldha*-Exon6-R	GAAGCCTCACCTGTCAACCA

SgRNAs

**Target gene**	**Target site**	**Cas9**		**sgRNA Sequence**	**PAM**
Ldha	Exon3	*Streptococcus pyogene*	sgRNA1	CCAAAAATTGTCTCCAGCAA	AGG
Ldha	Exon4	*Streptococcus pyogene*	sgRNA2	GCTGGTCATTATCACCGCGG	GGG
Ldha	Exon6	*Streptococcus pyogene*	sgRNA3	TGGAGTGGTGTGAATGTCGC	CGG
Ldha	Exon6	*Streptococcus pyogene*	sgRNA4	AAGTCTCTGAACCCGCAGCT	GGG
Ldha	Exon4	*Streptococcus pyogene*	sgRNA5	AGCTGGTCATTATCACCGCG	GGG
Ldha	Exon4	*Streptococcus pyogene*	sgRNA6	CTGCTCATCGTCTCAAACCC	AGG
Ldha	Exon6	*Streptococcus pyogene*	sgRNA7	GAAGTCTCTGAACCCGCAGC	TGG

Supplemental Table 2. Potential off-target sites and indel frequency for *Ldha* sgRNA.

Potential off-target sites predicted by Benchling software for the on-target sgRNA. Mismatched base pairs are labeled in red.

**Target ID**	**Sequence**	**PAM**	**Chromosome**	**Position**	**Direction**	**Mismatches**	**Indel frequency Reads (%)**	**Control Indel (%)**
Ldha	AGCTGGTCATTATCACCGCG	GGG	chr1	102904842	+	0	/	/
*Ldha*_off_1	AGCTGGTCATTATCACCGCG	GGG	chr6	79149970	−	0	0.44% (473/107377)	0.01% (2/15426)
*Ldha*_off_2	AGCTGGTCATTATCACCGCA	GGG	chr7	14762654	−	1	0.26% (275/107504)	0.30% (247/82237)
*Ldha*_off_3	AGCTGGTCATTATCACCGCA	GGG	chr7	14663350	−	1	0.17% (165/99164)	0.29% (228/79600)
*Ldha*_off_4	AGCTGGTCATTATCACGGCG	GGG	chr3	132807188	−	1	0.01% (10/112996)	0.02% (23/115667)
*Ldha*_off_5	AGCTGGTCATTATCACGGCG	GAG	chr3	37503615	−	1	0.01% (12/142794)	0.01% (10/133760)
*Ldha*_off_6	AGCTGGTCATTATCACAGCG	GGG	chrX	132572436	−	1	0.03% (30/102724)	0.13% (127/100245)
*Ldha*_off_7	AGCTGGTCATTATCACCTCG	GGG	chr8	33075496	+	1	0.26% (198/75208)	0.36% (255/70941)
*Ldha*_off_8	AGCTGGTTATTATCACCCCG	GGG	chr6	20949225	−	2	0.04% (47/111337)	0.01% (10/113929)

Ldha locus

#1

Sequence	Indel	%
TGTGACTGCAAACTCCAAGCTGGTCATTATCACCGCGGGGGCCCGTCAGCAAGAGGGAGAGAGCCGGCTC	WT	%
TGTGACTGCAAACTCCAAGCTGGTCATTATCACCCCGCGGGGGCCCGTCAGCAAGAGGGAGAGAGCCGGCT	+1bp	49.0
TGTGACTGCAAACTCCAAGCTGGTCATTATCA-CGCGGGGGCCCGTCAGCAAGAGGGAGAGAGCCGGCTC	-1bp	43.9
TGTGACTGCAAACTCCAAGCTG----------------------GTCAGCAAGAGGGAGAGAGCCGGCTC	-22bp	2.6
TGTGACTGCAAACTCCAAGCTGGTCA-------------------TCAGCAAGAGGGAGAGAGCCGGCTC	-19bp	0.9
TGTGACTGCAAACTCCAAGCTGGT---------------GGCCCGTCAGCAAGAGGGAGAGAGCCGGCTC	-15bp	0.8
TGTGACTGCAAACTCCAA----------------------GCCCGTCAGCAAGAGGGAGAGAGCCGGCTC	-22bp	0.6
TGTGACTGCAAACTCCAAGCTGGTCATTATCACACGCGGGGGCCCGTCAGCAAGAGGGAGAGAGCCGGCT	+1bp	0.6
TGTGACTGCAAACTCCAAGCT------------------GGCCCGTCAGCAAGAGGGAGAGAGCCGGCTC	-17bp	0.6
TGTGACTGCAAACTCCAAGCT-----------------------------------GAGAGAGCCGGCTC	-35bp	0.6
TGTGACTGCAAACTCCAAGCT---------------------------GCAAGAGGGAGAGAGCCGGCTC	-18bp	0.5

#2

Sequence	Indel	%
TGTGACTGCAAACTCCAAGCTGGTCATTATCACCGCGGGGGCCCGTCAGCAAGAGGGAGAGAGCCGGCTC	WT	%
TGTGACTGCAAACTCCAAGCTGGTCATTATCACCCCGCGGGGGCCCGTCAGCAAGAGGGAGAGAGCCGGCT	+1bp	49.3
TGTGACTGCAAACTCCAAGCTGGTCATTATCA-CGCGGGGGCCCGTCAGCAAGAGGGAGAGAGCCGGCTC	-1bp	43.9
--GGGAGAGAGCCGGCTC	-69bp	0.9
TGTGACTGCAAACTCCAAGCTGGTCATTAT---CGCGGGGGCCCGTCAGCAAGAGGGAGAGAGCCGGCTC	-3bp	0.9
TGTGACTGCAAACTCCAAGCTGGTCATTATCAC----------------CAAGAGGGAGAGAGCCGGCTC	-16bp	0.9
TGTGACTGCAAACTCCAAGCT------------------GGCCCGTCAGCAAGAGGGAGAGAGCCGGCTC	-18bp	0.9
TGTGACTGCAAACTCCAAGCT----------------GGGGCCCGTCAGCAAGAGGGAGAGAGCCGGCTC	-16bp	0.9
TGTGACTGCAAACTCCAAGCTG----------------------GTCAGCAAGAGGGAGAGAGCCGGCTC	-1bp	0.8
TGTGACTGCAAACTCCAAGCTGGTCA-------AGCTGGGGCCCGTCAGCAAGAGGGAGAGAGCCGGCTC	-7bp	0.7
TGTGACTGCAAACTCCAAGCTGGTCATTATCACACGCGGGGGCCCGTCAGCAAGAGGGAGAGAGCCGGCT	+1bp	0.7

#3

Sequence	Indel	%
TGTGACTGCAAACTCCAAGCTGGTCATTATCACCGCGGGGGCCCGTCAGCAAGAGGGAGAGAGCCGGCTC	WT	%
TGTGACTGCAAACTCCAAGCTGGTCATTATCA-CGCGGGGGCCCGTCAGCAAGAGGGAGAGAGCCGGCTC	-1bp	48.8
TGTGACTGCAAACTCCAAGCTGGTCATTATCACCCCGCGGGGGCCCGTCAGCAAGAGGGAGAGAGCCGGCT	+1bp	46.2
TGTGACTGCAAACTCCAAGCTGGTCATTAT---CGCGGGGGCCCGTCAGCAAGAGGGAGAGAGCCGGCTC	-3bp	0.8
TGTGACTGCAAACTC-------------------------------CAGCAAGAGGGAGAGAGCCGGCTC	-31bp	0.7
TGTGACTGCAAACTCC------------------------------CAGCAAGAGGGAGAGAGCCGGCTC	-30bp	0.7
TGTGACTGCAAACTCCAAGCTGGTCATTA----CGCGGGGGCCCGTCAGCAAGAGGGAGAGAGCCGGCTC	-4bp	0.6
TGTGACT---GAGAGCCGGCTC	-51bp	0.6
TGTGACTGCAAACTCCAAGCTGGTCATTAT--CCGCGGGGGCCCGTCAGCAAGAGGGAGAGAGCCGGCTC	-2bp	0.6
TGTGACTGCAAACTCCAAGCTGGTCATTATCACACGCGGGGGCCCGTCAGCAAGAGGGAGAGAGCCGGCT	+1bp	0.6
TGTGACTGCAAACTCCAAGCTGGTCATTATCACCACGCGGGGGCCCGTCAGCAAGAGGGAGAGAGCCGGC	+2bp	0.5

#4

Sequence	Indel	%
TGTGACTGCAAACTCCAAGCTGGTCATTATCACCGCGGGGGCCCGTCAGCAAGAGGGAGAGAGCCGGCTC	WT	%
TGTGACTGCAAACTCCAAGCTGGTCATTATCACCCCGCGGGGGCCCGTCAGCAAGAGGGAGAGAGCCGGCT	+1bp	56.8
TGTGACTGCAAACTCCAAGCTGGTCATTATCA-CGCGGGGGCCCGTCAGCAAGAGGGAGAGAGCCGGCTC	-1bp	30.9
TGTGACTGCAAACTCCAAGCTG----------------------GTCAGCAAGAGGGAGAGAGCCGGCTC	-22bp	4.1
TGTGACTGCAAACTCCAA----------------------GCCCGTCAGCAAGAGGGAGAGAGCCGGCTC	-22bp	1.6
TGTGACTGCAAACTCCAAGCT---------------GGGGGCCCGTCAGCAAGAGGGAGAGAGCCGGCTC	-15bp	1.3
TGTGACTGCAAACTCCAAGCTGGTCATTATCACACGCGGGGGCCCGTCAGCAAGAGGGAGAGAGCCGGCT	+1bp	1.1
---AGAGCCGGCTC	-66bp	1.1
TGT---GAGAGCCGGCTC	-55bp	1.1
TGTGACTGCAAACT----------------------------CCGTCAGCAAGAGGGAGAGAGCCGGCTC	-28bp	1.0
TGTGACTGCAAACTCCA------------------------------AGCAAGAGGGAGAGAGCCGGCTC	-30bp	1.0

#5

Sequence	Indel	%
TGTGACTGCAAACTCCAAGCTGGTCATTATCACCGCGGGGGCCCGTCAGCAAGAGGGAGAGAGCCGGCTC	WT	%
TGTGACTGCAAACTCCAAGCTGGTCATTATCACCCCGCGGGGGCCCGTCAGCAAGAGGGAGAGAGCCGGCT	+1bp	59.2
TGTGACTGCAAACTCCAAGCTGGTCATTATCA-CGCGGGGGCCCGTCAGCAAGAGGGAGAGAGCCGGCTC	-1bp	32.4
TGTGACTGCAAACTCCAAGCTG----------------------GTCAGCAAGAGGGAGAGAGCCGGCTC	-22bp	2.0
TGTGACTGCAAACTCCAAGCT------------------GGCCCGTCAGCAAGAGGGAGAGAGCCGGCTC	-18bp	1.2
TGTGACTGCAAACTCCAAGCTGGTCAT------------------TCAGCAAGAGGGAGAGAGCCGGCTC	-18bp	1.1
TGTGACTGCAAACTCCAAGCTGGTCATTATCACACGCGGGGGCCCGTCAGCAAGAGGGAGAGAGCCGGCT	+1bp	1.0
TGTGACTGCAAACTCCAAGCT----------------GGGGCCCGTCAGCAAGAGGGAGAGAGCCGGCTC	-16bp	0.8
TGTGACTGCAAACTCCAAGCTGGTCATTATCACCCCCGCGGGGGCCCGTCAGCAAGAGGGAGAGAGCCGGC	+2bp	0.8
TGTGACTGCAAACTCCAAGCTGGTCATTA----------------TCAGCAAGAGGGAGAGAGCCGGCTC	-16bp	0.8
TGTGACTGCAAACTCCAAGCTGGTCATTATCCCCCGCGGGGGCCCGTCAGCAAGAGGGAGAGAGCCGGCT	+1bp	0.7

#6

Sequence	Indel	%
TGTGACTGCAAACTCCAAGCTGGTCATTATCACCGCGGGGGCCCGTCAGCAAGAGGGAGAGAGCCGGCTC	WT	%
TGTGACTGCAAACTCCAAGCTGGTCATTATCACCCCGCGGGGGCCCGTCAGCAAGAGGGAGAGAGCCGGCT	+1bp	53.0
TGTGACTGCAAACTCCAAGCTGGTCATTATCA-CGCGGGGGCCCGTCAGCAAGAGGGAGAGAGCCGGCTC	-1bp	36.3
TGTGACTGCAAACTCCAAGCTG----------------------GTCAGCAAGAGGGAGAGAGCCGGCTC	-22bp	3.8
TGTGACTGCAAACTC----------------------------------CAAGAGGGAGAGAGCCGGCTC	-34bp	1.4
TGTGACTGCAAACTCCAAGCTGGT-----------------CCCGTCAGCAAGAGGGAGAGAGCCGGCTC	-17bp	1.2
TGTGACTGCAAACTCCAAGCTGGTCATTAT---CGCGGGGGCCCGTCAGCAAGAGGGAGAGAGCCGGCTC	-3bp	0.9
TGTGACTGCAAACT---------------------------CCCGTCAGCAAGAGGGAGAGAGCCGGCTC	-27bp	0.9
TGTGACTGCAAACTCCAA----------------------GCCCGTCAGCAAGAGGGAGAGAGCCGGCTC	-22bp	0.9
TGTGACTGCAAACTCCAAGCTGGTCATTATCACCCCCGCGGGGGCCCGTCAGCAAGAGGGAGAGAGCCGGC	+2bp	0.8
TGTGACTGCA---GGAGAGAGCCGGCTC	-45bp	0.7

Supplementary Figure 1. Top 10 indels of the *Ldha* gene generated by CRISPR/Cas9. Deep sequencing was performed on the DNA from livers of 6 PH1 rats treated with the AAV-CRISPR/Cas9 system one month after therapeutic administration. Indel sizes and frequency are listed on the right. The PAM sequence is in green and the nucleotide substitutions are indicated by the blue characters. Red letters indicate inserted nucleotides and black dashes deleted nucleotides.

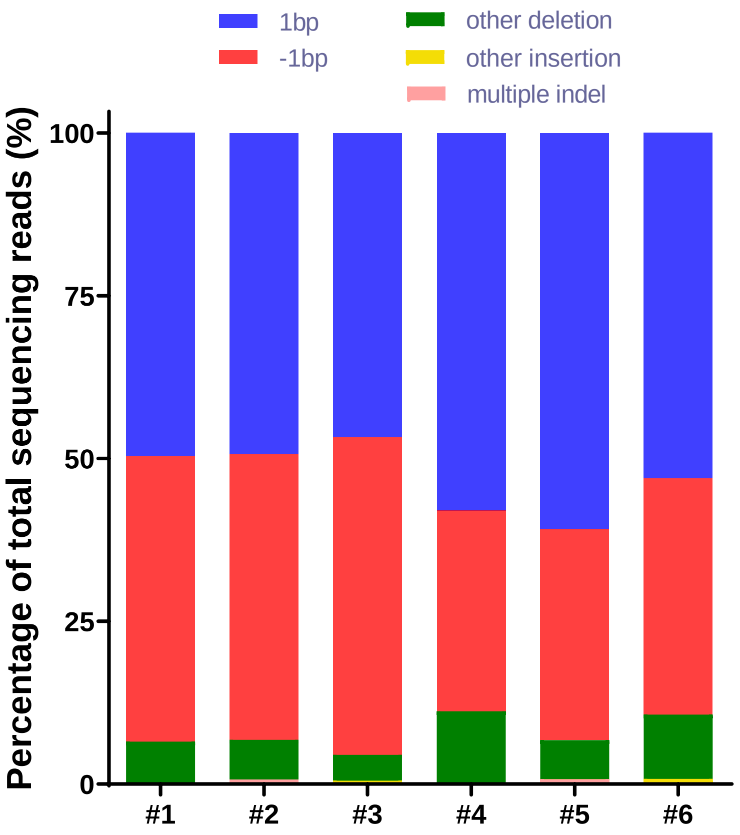

Supplementary Figure 2. Characterization of *Ldha* gene generated by CRISPR/Cas9. Characterization of the indels of each animal treated with AAV-Cas9 and AAV-*Ldha*-sgRNA according to the size.

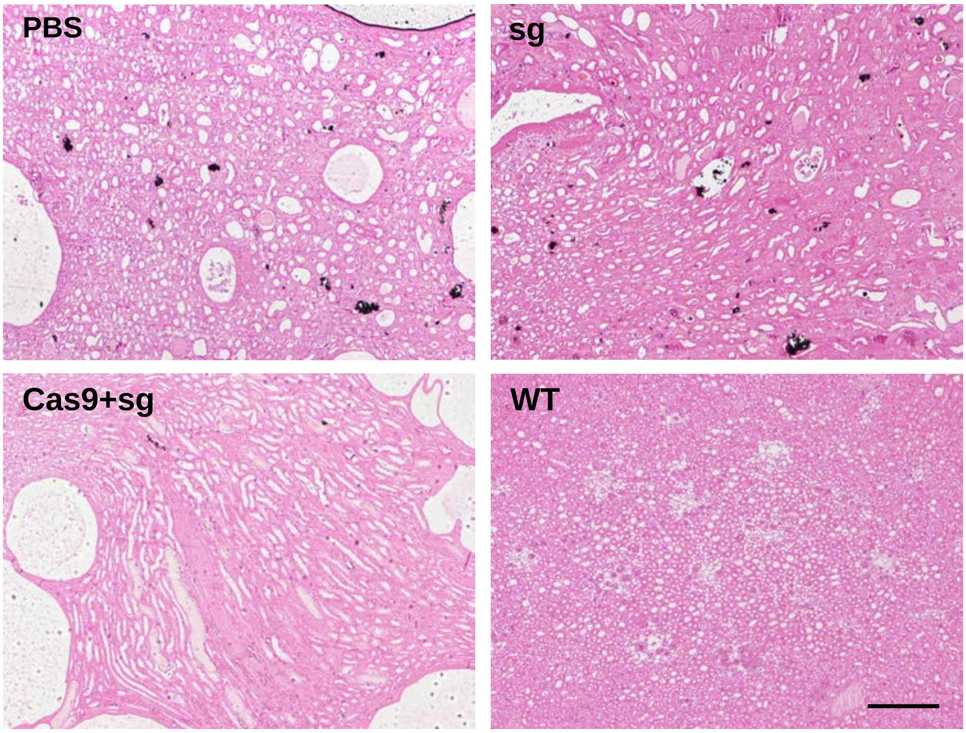

Supplementary Figure 3. Pizzolato staining of renal medulla.
Representative Pizzolato staining of renal medulla sections from *Agxt*^{D205N} rats sacrificed 5 months after treatment with PBS, AAV-sgRNA, or dual AAV-Cas9 and AAV-sgRNA and 1 month after 0.5% ethylene glycol challenge. Renal medulla sections of WT rats of the same age were used as controls. CaOx deposits were stained in black. Scale bar, 200 μm.

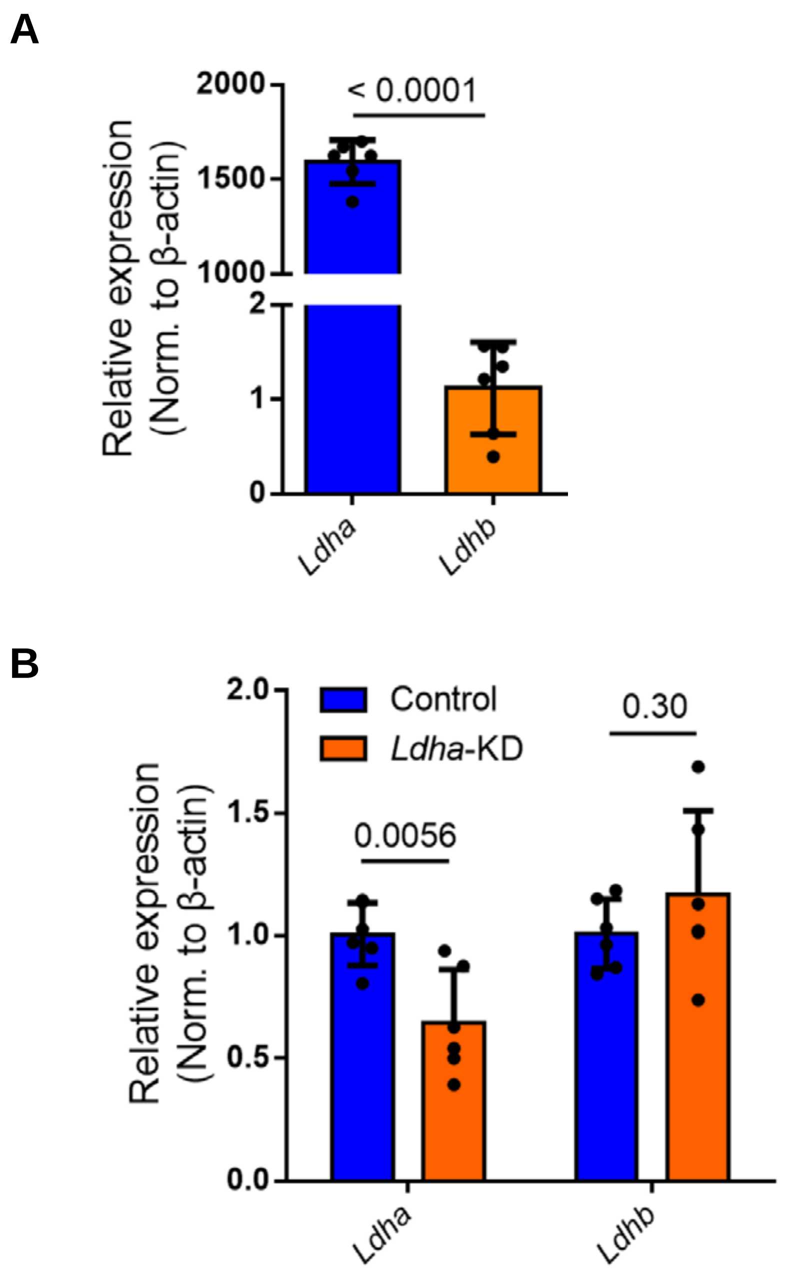

Supplementary Figure 4. Expression of liver *Ldha* and *Ldhb* in *Agxt*^{D205N} rats.

A. Relative mRNA expression levels of *Ldha* and *Ldhb* gene in liver tissue from 12-week-old *Agxt*^{D205N} rats. N = 6 in each group. B. Quantification of *Ldha* and *Ldhb* mRNA levels in liver tissue from *Agxt*^{D205N} rats (control) and *Agxt*^{D205N} rats with *Ldha*-knockdown (*Ldha*-KD). N = 6 in each group. Results: mean ± SD. Significant *p* values are denoted above the bars. KD, knockdown. Norm., normalized.
